# Facilitators and barriers to the implementation of the Girls' Iron Folate Tablet Supplementation program among adolescent girls in the Bono Region: a cross-sectional survey

**DOI:** 10.11604/pamj.2025.50.57.44319

**Published:** 2025-02-20

**Authors:** Ba-Etilayoo Atinga, Albert Henyo, Christiana Ayamga, Moses Issah Mensah, Bismark Gyan, Helina Michelle Sarfo, Dione Kwakye, Desmond Kuupiel

**Affiliations:** 1 Department of Nursing, School of Sciences, University of Energy and Natural Resources, Sunyani, Bono Region, Ghana,; 2 Department of Maternal and Child Health, School of Nursing and Midwifery, College of Health Sciences, University of Ghana, Legon, Ghana,; 3 Department of Public Health, School of Nursing and Public Health, University of KwaZulu-Natal, Durban, 4001, South Africa,; 4 Faculty of Health Sciences, Durban University of Technology, Durban, 4001, South Africa

**Keywords:** Anaemia, adolescent girls, iron folic acid, GIFT program, Bono Region, cross-sectional survey

## Abstract

**Introduction:**

iron supplementation is one of the primary cornerstone treatments for reducing anaemia in pregnant women, and its adoption is important for reducing anaemia to tolerable levels. About 33% of women of reproductive age worldwide are anaemic. As a result, women are more likely to enter pregnancy with less-than-optimal iron stores, which may have negative consequences for them and their offspring, including an increased risk of miscarriage, stillbirth, prematurity, and low birth weight, as well as impaired physical and neurological development. This study aims to determine the challenges of implementing the Girl Iron Folate Tablet Supplementation (GIFTS) program among adolescent girls.

**Methods:**

this was a cross-sectional descriptive study involving females at Fiapre Methodist and Saint Thomas Junior High Schools at Fiapre in the Sunyani West Municipality. Data were collected on socio-demographic characteristics, knowledge on anaemia, knowledge on GIFTS program barriers and facilitators of the GIFTS program using a face-to-face semi-structured questionnaire. Descriptive statistics were done to determine knowledge on anaemia and GIFTS program while a Chi-square analysis was performed to determine factors influencing iron and folic acid (IFA) utilization.

**Results:**

a total of 138 participants were included in this study. Knowledge and compliance with the IFAS program among adolescent girls were good (84.1%). Lack of information, opposition from their family and fear of side effects of the tablets were seen as the major barriers to compliance. The Chi-square test of independence revealed no significant association between socio-demographic characteristics. Iron fortification of foods and/or changing composition of iron preparation to avoid side effects may also be considered in the future.

**Conclusion:**

knowledge and compliance with the IFAS program among adolescent girls were good. Majority of the respondents (70.6%) reported that they stopped taking the supplements temporary after they encountered problems as a measure. Including community leaders religious and community leaders, provision of education and counselling and reducing long waiting hours were to be the major promoting factors to the implementation of the WIFA program.

## Introduction

Anaemia is a medical condition characterised by a deficiency in red blood cells or haemoglobin concentration, particularly prevalent among young children, pregnant and postpartum women, and menstruating adolescent girls and women [1-3]. Insufficient haemoglobin leads to reduced oxygen transportation to tissues and organs [1-3]. Severe cases can result in cognitive and motor developmental issues in children and complications during pregnancy [1-3]. Contributing factors include poor nutrition (including intake of low-iron meals), infections, chronic illnesses, heavy menstruation, pregnancy complications, and genetic predisposition, with iron deficiency being a common cause [1-3].

Anaemia represents a significant global public health challenge, impacting a substantial portion of the population [1,4]. Roughly half a billion women aged 15-49 and 269 million children aged 6-59 months worldwide are affected [1,4]. In 2019, anaemia affected approximately 30% of non-pregnant women (539 million) and 37% of pregnant women (32 million) in these age groups [1,4]. Particularly acute in the WHO Regions of Africa and South-East Asia, the burden is staggering, with an estimated 106 million women and 103 million children affected by anaemia in Africa, and 244 million women and 83 million children affected in South-East Asia [1,4]. The United Nations International Child Education Fund reports also suggested that 33% of women of reproductive age worldwide are anaemic [3]. As a result, women are more likely to enter pregnancy with less-than-optimal iron stores, which may have negative consequences for them and their offspring, including an increased risk of miscarriage, stillbirth, prematurity, and low birth weight, as well as impaired physical and neurological development [4].

In Ghana, the Ghana Demographic and Health Survey (GDHS) Report suggests more than 4 out of every 10 women (42%) are anaemic, with the Upper East Region accounting for 39.6 percent [5]. The proportion of women with mild anaemia is greater in rural regions (44%) than in urban areas (41%). In Ghana, about 32.2%, 9.8% and 0.4% of the population respectively, have mild, moderate, or severe anaemia [6]. Moreover, the 2017 Ghana Micronutrient Survey indicated that the countrywide prevalence of anaemia among non-pregnant teenage females aged 15-19 years is 26.4% [7]. In the Bono Region of Ghana, a study estimated the prevalence rate of anaemia to be about 40.8% among the general population in the Sunyani Municipal area [8].

While the causes of anaemia are complex, it is believed that 50% of anaemia cases are caused by iron deficiency [2]. Evidence shows iron deficiency anaemia affects 38.2% of pregnant women worldwide and 46.3% of pregnant women in Africa [9]. To this end, the World Health Organization suggests an approach that includes weekly iron supplementation [10] since it has been shown to improve anaemia and raise iron needs in teenage females who menstruate [11], and reduce the incidence of anaemia in adolescents [12]. In Ghana, however, several intervention schemes have been implemented to combat anaemia among pregnant women. Among these interventions include the distribution of Long-Lasting Insecticide-Treated Nets (LLIN), national deworming exercises, the use of Sulphadoxine-pyramethine (SP) as malaria prophylaxis, and the use of iron supplements (ferrous sulphate, folic acid, and multivitamins) [5]. Despite the above-mentioned continuing measures, the country, especially the Bono Region, continues to see worrisome rates of anaemia [8]. The level of compliance with iron supplement consumption in Ghana, and particularly in the study district, has received little attention. This study assesses the challenges of implementing the Girls Iron Folate Tablet Supplementation program among adolescent girls in Fiapre, Bono Region.

## Methods

### Study design

This research employed a cross-sectional descriptive study design involving adolescents aged 10 to 19 years of schooling at Fiapre Methodist Junior High School and Saint Thomas Junior High School at Fiapre, Sunyani West Municipal, Bono Region.

### Study setting

Fiapre, Sunyani West is one of the municipalities in the Bono region of Ghana. Sunyani West Municipal was formally part of the Sunyani Municipal until the northwest part was separated to create Sunyani Municipal on 1^st^ November 2007 and has Odumase as its capital town. It shares boundaries with Tain, Berekum, Sunyani Municipality, Techiman, Wenchi, Dormaa East and Tano North. According to the Ministry of Finance budget estimation for 2020, the size of Sunyani West is 1,059.33 square kilometers. There are 133 settlements, 4 of which are urban; Fiapre, Odumase, Chiraa and Nsuatre. Fiapre has one of the best female secondary schools in the region known as, Notre Dame Senior High School. It also hosts Holy Spirit School, a Catholic School aimed at holistic education. Fiapre Methodist as well as Saint Thomas Junior High School among others are all located in Fiapre. Agriculture is the main occupation for the people in the town while teaching, civil service and private businesses also play a very big role.

### Participants

Potentially eligible: all female students aged 10-19 years from the two selected junior high schools in Fiapre. The head of the schools provided a list of all students in the selected schools. Researchers located and contacted potential volunteers to schedule an initial meeting. Students who met the inclusion criteria and were interested in participating were provided with an information sheet and a consent form. A total of 138 participants who consented and met the criteria were included in the study. Data collection was conducted at a single point in time. All 138 participants were included in the data analysis.

### Variables

Outcome variables: utilization of IFA tablets (measured as whether participants have consumed IFA tablets before), knowledge about anaemia and GIFTS program, perceived benefits and side effects of IFA tablets predictors (age, level, religious affiliation).

### Data sources/measurement

A semi-structured questionnaire was developed and used to collect data. The questionnaire included sections on socio-demographic characteristics, knowledge about anaemia, knowledge about the GIFTS program, and barriers and facilitators for the program. Data collection was conducted at field level by medical staff using a paper-based patient data form.

Data from previous studies (e.g. UNICEF and WHO reports) were used to provide context and compare findings. This helps in understanding the broader picture of iron supplementation efforts. The data collected via questionnaires was entered into SPSS, for analysis. Data was coded systematically to facilitate analysis. Any discrepancies or missing data were addressed through imputation methods.

### Bias

A p-value of less than 0.05 was used to declare a statistically significant presence of publication bias. Missing data were addressed using multiple imputations to avoid bias from excluding incomplete cases. Subgroup analyses were conducted for different age groups and schools to identify biases specific to these subpopulations. The study used validated questionnaires to measure knowledge and compliance, thereby minimizing measurement error and associated bias. Statistical analyses were performed using SPSS version 26, ensuring standardized and reliable computational methods.

### Sample size determination

In this study, sample size determination was based on an estimated proportion of adolescent girls who consumed IFA tablets (90% consumption rate in Northern and Volta regions of Ghana) [13], a 95% confidence level, 5% margin of error. Sample size calculation was done to identify the minimum number of participants to be used. It was based on the Cochrane formula, which states:

$$N=\frac{Z^{2}xP\left(1-P\right)}{E^{2}}$$

Where, N= minimum sample size, N=?, Z= Confidence level, Z =95%, P= Estimated proportion of adolescent girls who consumed IFA tablets, 90% consumption rate in Northern and Volta regions of Ghana according to [13]; E= Margin of error= 5%; N= [(1.96) ^2^ × 0.90(1-(1.96) ^2^ × 0.90(1-0.90)] ÷ (0.05)^2^; = (3.8416 × 0.09) / 0.0025; = 0.345744 / 0.0025 = 138.2976; = 138 participants. This gave a sample size of 138 adolescent girls aged 10-19.

### Sampling procedure

A multi-stage sampling method was used for this study. First, a purposive sampling technique was employed to select the two schools. Next, systematic sampling technique was employed to sample the students (half of the respondents (50%) were students at Saint Thomas J.H.S, whilst the other half (50%) schooled at Fiapre Methodist J.H.S); that is, every third female in each class was selected.

### Data collection

The University of Energy and Natural Resources' institutional review board received a copy of this application and provided the researchers with ethical clearance and approval to enlist study participants. The researchers delivered the credentials and an introductory letter from the University of Energy and Natural Resources-Department of Nursing to Ghana Education Service who gave a letter to the head of the selected schools; Fiapre Methodist Junior High School and Saint Thomas Junior High School. This was done to introduce the researcher and gain official approval and consent for the study site and participants. The head of the schools assisted the researchers by providing a list of all students in the selected schools. After receiving approval, the researchers located and contacted potential volunteers to schedule an initial meeting.

All interested participants and the researcher met on the agreed-upon day to discuss the study's relevance. This ensured that participants had a better knowledge of the study. Those who satisfied the criteria and expressed an interest in participating received an information sheet and a consent form to sign. A structured questionnaire was then provided to participants on a face-to-face basis to complete. The questionnaire consisted of 4 sections, namely the socio-demographic characteristics of participants, knowledge of the subjects on anaemia, knowledge, and perception of adolescent girls of the GIFTS program and finally barriers and facilitators of the GIFTS program.

### Statistical methods

This study employed descriptive statistics to summarize the socio-demographic characteristics of the participants, their knowledge of anaemia and the GIFTS program, and their utilization of IFA tablets. Frequencies and percentages were used for categorical variables, while means and standard deviations were reported for continuous variables, such as age.

Any missing data were addressed through pairwise deletion, meaning that cases with missing values were excluded from specific analyses, but included in others where data were available.

Potential confounders, such as age, grade level, and school attended, were identified. Chi-square tests were used to assess the relationship between these variables and the utilization of IFA tablets.

Subgroup analyses were conducted to compare IFA tablet utilization across different age groups, grade levels, and religious affiliations. Chi-square tests were also used to examine these subgroup differences.

### Sensitivity analyses

Sensitivity analyses were performed to test the robustness of the findings. Analyses were repeated excluding participants with incomplete data to ensure that the results were not unduly influenced by missing values. All statistical analyses were conducted using SPSS (Statistical Package for the Social Sciences) version 26.0. This software facilitated the calculation of descriptive statistics, chi-square tests, and the handling of missing data.

### Data analysis and interpretation

The data was analysed using SPSS version 26 software. Cross-tabulation was used to generate frequencies and percentages in tables and charts. Afterwards, bivariate analysis was done using the Chi-square test to test the association between the dependent and independent variables. The association is considered significant when the p-value is less than 0.05. Descriptive statistics such as frequency distribution, and measures of central tendency were used.

Knowledge was assessed by a three-item scale. A score of one was given if a respondent chose a correct answer. A score of zero was given in cases where a respondent chose a wrong answer or “do not know” option. The total knowledge score was calculated by adding the score of each respondent with a maximum score of 10. Total knowledge score was expressed as mean. Afterwards, the total knowledge score was categorized into good and poor knowledge levels based on Bloom´s cut-off point which indicates a score of =80% to be good knowledge and <80% as poor knowledge.

### Ethics approval and consent to participate

To ensure that these principles are being observed, the researchers sought ethical clearance from the Research Ethics Committee at the University of Energy and Natural Resources. Permission was also sought from the authorities of the selected schools before the research started. Informed consent was obtained from the participants. Consent forms were issued to participants after the purpose and objectives of the study were explained to them. Participants were given full information about the research (objectives, risks and benefits, responsibilities of the researcher and measures to ensure participants are not harmed). Participants were further informed that taking part was voluntary and that they had the right to withdraw from the study without any penalty. All agreements made were honoured and all outlined procedures were adhered to. Moreover, anonymity and confidentiality were ensured, and names or any other identifying data of participants were not used.

## Results

### Socio-demographic characteristics of respondents

Table 1 represents the socio-demographic data of respondents of this study. Of the 138 respondents, most were J.H.S two students (n= 59, 42.8%), and Christians (n= 114, 82.6%). The average age of the study´s respondents was 12 years.

**Table 1:** socio-demographic characteristics of respondents

Frequency (%)	
**School**	
Saint Thomas	69 (50.0)
Fiapre Methodist	69 (50.0)
**Current level**	
Form 1	33(23.9)
Form 2	59 (42.8)
Form 3	46 (33.3)
**Religious affiliation**	
Islamic	24 (17.40)
Christianity	114 (82.6)
Traditionalist	0 (0)

### Respondents´ knowledge of anaemia and iron and folic acid programme

Of the 138 respondents, 116 (84.1%) respondents showed good knowledge, while 22 (15.9%) showed poor knowledge of anaemia. While 99.3% of the total 138 respondents indicated that they have heard about the weekly iron folate supplementation program before, a few (0.7%) indicated that they have never heard about it. About (93.5) of adolescent girls stated that the IFA supplementation protects against anaemia, and about (98%) indicated that the program helps in perform better physically. More than half (71%) of the respondents said the supplements must be taken four times within a month. Nearly all (97%) of the adolescent girls indicated that the supplements help improve their learning capacity. About (80%) indicated that the appropriate age to start the program is ten years (Table 2).

**Table 2:** awareness and patronage of the IFA program

Variables	Frequency	Percentage %
**Knowledge level**		
Low	22	15.9%
High	116	84.1%
**Heard of WIFA**		
YES	137	99.3%
NO	1	0.7%
**IFA protects against anaemia**		
NO	9	6.5%
YES	129	93.5%
**IFA ensures better physical performance**		
NO	2	1.4%
YES	136	98.6%
**IFA ensures improved learning capacity**		
NO	4	2.9%
YES	134	97.1%
**Number of times IFA tablets should be taken**		
No idea/once/twice/3 times	40	29.0%
4 times	98	71.0%
**Appropriate age to start taking IFA tablets**		
15 years/18 years and above	27	19.6%
10 years	111	80.4%

### Source of information

**Figure 1:**
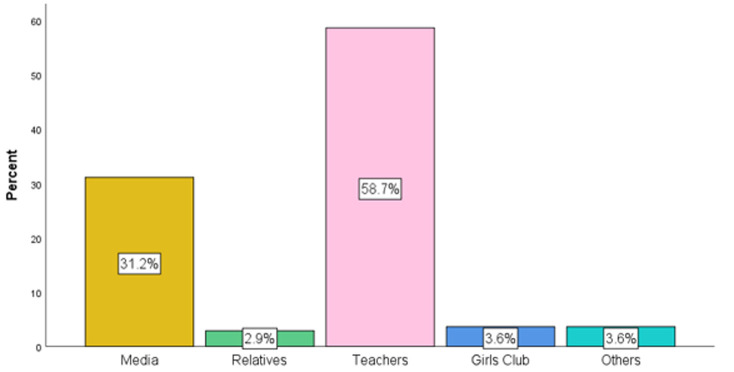
source of information

With regards to the source of information on the IFA program, teachers were the sources for most of the 138 respondents (n = 81, 58.7%), whilst relatives were the source for a few students (n= 4, 2.9%) (Figure 1).

### Utilization of the iron and folic acid supplementation

**Figure 2:**
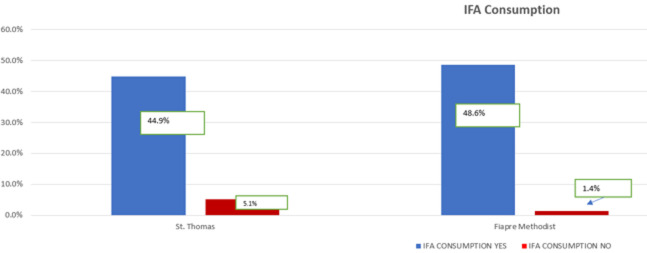
utilization of the IFA supplementation

Almost all of the 138 respondents (n= 129, 93.5%) had consumed the IFA tablets before, with just a few (n= 9, 6.5%) indicating that they had never consumed the tablets before. IFA tablet consumption was high among adolescent girls at Fiapre Methodist (48.6% out of 50 respondents) compared to its use at Saint Thomas J.H.S (44.9 out of 50 students) (Figure 2).

### Preferences and challenges to the implementation of the iron and folic acid program

Majority (87%) of the respondents have never discussed the IFA program with their parents. With those who have discussed with their parents, more than three-quarters (83.3%) preferred informing their mothers to informing their fathers (16.7%), with none of the respondents discussing with their other relatives. With regards to the response of their parents, nearly half of the respondents (44.4%) indicated that the outcome was encouraging. More than half of the respondents (63%) indicated that they have not encountered any problems since the utilization of the tablets whilst minority (37%) stated that they, have at some point encountered some problems. For those who have encountered some problems, majority (47.1%) stated that they had increased menstrual flow, 39.2% indicated that they had heartburns and 13.7% made it known that they gained more weight after taking the tablets. Majority of the respondents (70.6%) reported that they stopped taking the supplements temporarily after they encountered problems, about 15% said they did nothing about it whilst 13.7% of the respondents said they reported to a healthcare provider the problems they encountered. More than three-quarters (81.4%) said the side effects never prevented them from taking the tablets whilst some (18.6%) of the adolescent girls stated that indeed the problems they encountered prevented them from taking the supplements. More than half (68.1%) indicated that the program consumes too much time whilst 31.9% reported the opposite.

### Relationship between socio-demographic characteristics and iron and folic acid tablet utilization

Table 3 displays the relationship between socio-demographic characteristics and the utilization of iron and folic acid (IFA) tablets. It indicates that there are significant differences in IFA tablet consumption based on age group (χ2= 23.9, p= .015), with a higher proportion of individuals aged 15-19 reporting consumption compared to those aged 10-14. However, no significant differences were found based on school attended (?2= 2.972, p= .085), education level (χ2= 1.029, p= .598), or religion (χ2 = 0.264, p= .607).

**Table 3:** relationship between socio-demographic characteristics and IFA tablet utilization

		Ever consumed IFA tablet		Total	X^2^ value	Df	P-value
		YES	NO				
Age group	10-14	31 (22.5%)	2 (1.4%)	23.9%	.015	1	0.902
	15-19	98 (71.0%)	7 (5.1%)	76.1%			
School	Saint Thomas	62 (44.9%)	7 (5.1%)	50.0%	2.972	1	0.085
	Fiapre Methodist	67 (48.6%)	2 (1.4%)	50.0%			
Level	Form 1	32 (23.2%)	1 (0.7%)	23.9%	1.029	2	0.598
	Form 2	54 (39.1%)	5 (3.6%)	42.8%			
	Form 3	43 (31.2%)	3 (2.2%)	33.3%			
Religion	Islamic	23 (16.7%)	1 (0.7%)	17.4%	.264	1	0.607
	Christianity	106 (76.8%)	8 (5.8%)	82.6%			

## Discussion

### Knowledge on anaemia

The study revealed a good knowledge level (84.1%) among adolescent girls in the selected schools. The result is slightly higher than a study in Ghana by Unicef [13] which stated that more than half (63.8%) of girls in JHS and SHS collectively had ever heard of anaemia. On the other hand, it is at variance with the results of a study in the Northern region of Ghana among school-going girls, where less than half (49%) have some knowledge of anaemia at a baseline [13]. This study is also contrary to [14] which stated in a study in Ethiopia that less than half of the girls heard the term anaemia, and about one third knew the relationship between anaemia and the intake of iron-rich foods.

### Awareness and utilization of the iron and folic acid program

Nearly all (99.3%) of the respondents in this study indicated that they had heard about the weekly iron folate supplementation program before, where the majority (58.7%) indicated their teachers as the major source of information on the IFA program. This is agreed by a study which revealed that more than two-thirds of adolescents were aware of the program and more than three-quarters (75%) accessed information related to WIFAS from their school teachers followed by relatives (8.9%) [12]. This is contrary to a study in Tamale, which revealed that about one-third (35.1%) of the adolescent girls were found to have good knowledge of the IFAS program. Good level of awareness of the IFAS program might be due to adolescent girls receiving adequate education on the IFAS program from their teachers [15].

Findings in this study showed that almost all (93.5%) of the respondents had consumed the IFA tablets before. This is similar to a study by Unicef [13], which stated that about 1329 students (94.8%) had ever consumed IFA tablets given by a teacher at school. However, the result is contrary to a different study where only about 25% of children were consuming IFA tablets [16]. This is in variance with a study that revealed the overall compliance with the IFAS to be (26.2%) [15].

### Barriers to the implementation of the iron and folic acid program

The majority of the respondents (70.6%) in this study reported that they stopped taking the supplements temporary after they encountered problems. This is similar to a study which revealed that about one-quarter of the respondents who stopped taking the tablets indicated stomach pain, fear of side effects, bad taste and black coloured stool to be the reasons for the discontinuation [17]. In this study, about 13% of the respondents had discussed the IFA program with their parents. This disagrees with a study by Desta *et al*. [18]. This showed that majority of adolescent girls who are included in the study discussed with their families on their status of taking IFA [18].

The minority (37%) of adolescents stated that they have at some point encountered some problems in taking the tablets which is higher compared to a study by Gosdin *et al*. [19] indicating that about (9%) have ever encountered problems. For those who have encountered some problems, majority (47.1%) stated that they had increased menstrual flow as the major problem which agrees with a study that reported that majority (27%) indicated heartburn to be the main problem encountered [19]. The majority of the respondents (70.6%) reported that they stopped taking the supplements temporary after they encountered problems as a measure. In another study, the majority visited a healthcare provider after encountering a problem [20].

The majority (71%) perceived that lack of information, opposition from their family (70.3%) and fear of side effects of the tablets (65.9%) were the barriers to the implementation of the program. These findings were supported by a study conducted by [21] where 12% of study population discontinued IFA tablets due to side effects. The finding is also similar to a study where fear of side effects of IFA tablets and lack of awareness on the program were the major barriers. Thus, participants could enumerate a different set of factors that acted as barriers for IFA consumption in the study area [18].

With regards to the promoting factors, respondents indicated including community leaders (63.8%), religious leaders (51.4%), community leaders (59.4%), education and counselling (52.9%) and reducing long waiting hours (51.4%) to be the major promoting factors to the implementation of the WIFA program. This is similar to a study by Mulugeta *et al*. [22] which stated in research done in Ethiopia that many players, including local government offices, family members, and community leaders, might play a role in promoting iron supplementation initiatives for teenage females. Similarly, as family members understood the goal of the supplementation programme, they became supportive and played an important role in reminding women to take the supplements [23].

### Relationship between socio-demographic characteristics and iron and folic acid tablet utilization

The chi-square test of independence in this study revealed no significant association between socio-demographic characteristics. This is contrary to a study where socio-demographic characteristics such as adolescents´ age and level were associated with whether girls ever consumed an IFA tablet [23]. In another study, the level of education and occupation of mothers of adolescent girls, awareness of anaemia, and good knowledge of anaemia and of the IFAS program were significant predictors of compliance with the IFAS [15].

### Implications of the Girl Iron Folate Tablet Supplementation study

The study reveals that iron-folic acid supplementation can significantly improve haemoglobin levels and reduce anaemia among adolescent girls in the Bono Region of Ghana, thereby reducing long-term health complications and potentially breaking the poverty cycle by enhancing cognitive function and school performance. Finally, the findings of this study support the integration of iron-folic acid supplementation into public health programs targeting adolescent girls, especially in regions where anaemia prevalence is high.

### Strength and limitation

The research demonstrates a strong understanding of the factors influencing adolescent girls' compliance with the IFAS program. It effectively identifies key barriers such as lack of information, familial opposition, and concerns about side effects, backed by compelling evidence from the respondents. Additionally, the study provides actionable recommendations for improving program implementation, including involvement of community and religious leaders, provision of education and counselling, and addressing logistical challenges like long waiting times. Despite these strengths, this study has limitations. Firstly, the current sample was not representative of the Ghanaian population, hence a large sample size to enhance the study's statistical power and generalizability of findings to the target population of adolescent girls in Ghana needs to be addressed. In addition, the study may not fully account for contextual factors such as dietary habits, socio-economic status, and access to healthcare, which could confound the relationship between supplementation and health outcomes. Nonetheless, this study potentially can inform effective interventions to improve GIFTS program in this study area and similar geographic settings.

## Conclusion

This study indicates in clear terms the need to augment the knowledge and compliance with the IFAS program among adolescent girls. Lack of information, opposition from their family and fear of side effects of the tablets were seen as the major barriers to compliance. For instance, Majority of the respondents (70.6%) reported that they stopped taking the supplements temporarily after they encountered problems as a measure. Including community leaders religious and community leaders, provision of education and counselling and reducing long waiting hours were to be the major promoting factors to the implementation of the WIFA program.

### What is known about this topic

- The GIFTS program combats iron deficiency anaemia in adolescent girls by providing iron and folic acid tablets, implemented in schools and communities through health and education sector collaboration;
- Challenges of the GIFTS program include supply chain management and cultural misconceptions hindering program effectiveness;
- Success factors include effective training of teachers and healthcare workers, regular monitoring and community engagement, and supportive policies and funding.

### What this study adds

- This study provides detailed, localised insights into the specific challenges and facilitators of the GIFTS program among adolescent girls in the Bono Region, adding valuable data that addresses regional implementation issues often overlooked in broader studies;
- By focusing on the perspectives of adolescent girls and key stakeholders such as teachers and healthcare workers, this study enriches the understanding of the multifaceted barriers to iron and folate supplementation, including socio-cultural and educational factors;
- This study offers targeted recommendations for improving the GIFTS program's effectiveness, such as enhancing awareness campaigns, improving supply chain management, and fostering community engagement, thereby providing actionable insights for policymakers and practitioners.

## References

[1] Gebreegziabher T, Sidibe S (2023). Prevalence and contributing factors of anaemia among children aged 6-24 months and 25-59 months in Mali. J Nutr Sci.

[2] Azinge IE, Ogunyemi A, Ogamba CF, Jimoh RO (2023). Prevalence of anemia and associated factors among adults in a select population in Lagos, Southwest Nigeria. J Public Health Afr.

[3] Zegeye B, Anyiam FE, Ahinkorah BO, Ameyaw EK, Budu E, Seidu AA (2021). Prevalence of anemia and its associated factors among married women in 19 sub-Saharan African countries. Arch Public Health.

[4] Abu-Ouf NM, Jan MM (2015). The impact of maternal iron deficiency and iron deficiency anemia on child's health. Saudi Med J.

[5] Ghana Statistical Service (2024). Ghana Demographic and Health Survey 2022. Ghana Statistical Service.

[6] Wemakor A, Kwaako M, Abdul-Rahman A (2023). Nutritional, health and socio-demographic determinants of anaemia in adolescent girls in Kumbungu District, Ghana. BMC nutrition.

[7] Rohner F, Tanumihardjo S, Steiner-Asiedu M, Williams TN, Wirth JP, Petry N Revised survey protocol Ghana Micronutrient Survey 2017 (GMS 2017).

[8] Anlaakuu P, Anto F (2017). Anaemia in pregnancy and associated factors: a cross sectional study of antenatal attendants at the Sunyani Municipal Hospital, Ghana. BMC Res Notes.

[9] Zafar DA (2021). Iron Supplementation in Pregnancy-The Most Needed But the Most Neglected Element of Antena-tal Care. Archives of Nutrition and Public Health.

[10] Roche ML, Samson KLI, Green TJ, Karakochuk CD, Martinez H (2021). Perspective: Weekly Iron and Folic Acid Supplementation (WIFAS): A Critical Review and Rationale for Inclusion in the Essential Medicines List to Accelerate Anemia and Neural Tube Defects Reduction. Adv Nutr.

[11] Fernández-Gaxiola AC, Neufeld LM, García-Guerra A (2024). Considerations for Correction of Micronutrient Deficiencies Through Supplementation in Pregnant Women and Children Under-5 in Latin America. Food Nutr Bull.

[12] Gosdin L, Sharma AJ, Tripp K, Amoaful EF, Mahama AB, Selenje L (2021). A School-Based Weekly Iron and Folic Acid Supplementation Program Effectively Reduces Anemia in a Prospective Cohort of Ghanaian Adolescent Girls. J Nutr.

[13] Unicef Ghana Health Service, Ghana Education Service (2019). The Girls’ Iron-Folic Acid Tablet Supplementation (GIFTS) Programme: An Integrated School-Based Nutrition and Health Intervention. Baseline and Follow-On Impact Evaluation in Northern and Volta Regions, Republic Of Ghana, 2017-2018.Unicef.

[14] Gebreyesus SH, Endris BS, Beyene GT, Farah AM, Elias F, Bekele HN (2019). Anaemia among adolescent girls in three districts in Ethiopia. BMC public health.

[15] Dubik SD, Amegah KE, Alhassan A, Mornah LN, Fiagbe L (2019). Compliance with weekly iron and folic acid supplementation and its associated factors among adolescent girls in Tamale Metropolis of Ghana. Journal of nutrition and metabolism.

[16] Priya SH, Datta SS, Bahurupi YA, Narayan KA, Nishanthini N, Ramya MR (2016). Factors influencing weekly iron folic acid supplementation programme among school children: Where to focus our attention?. Saudi Journal for Health Sciences.

[17] Harris LA, Horn J, Kissous-Hunt M, Magnus L, Quigley EMM (2017). The Better Understanding and Recognition of the Disconnects, Experiences, and Needs of Patients with Chronic Idiopathic Constipation (BURDEN-CIC) Study: Results of an Online Questionnaire. Adv Ther.

[18] Desta M, Kassie B, Chanie H, Mulugeta H, Yirga T, Temesgen H (2019). Adherence of iron and folic acid supplementation and determinants among pregnant women in Ethiopia: a systematic review and meta-analysis. Reprod Health.

[19] Gosdin L, Sharma AJ, Tripp K, Amoaful EF, Mahama AB, Selenje L (2020). Barriers to and Facilitators of Iron and Folic Acid Supplementation within a School-Based Integrated Nutrition and Health Promotion Program among Ghanaian Adolescent Girls. Curr Dev Nutr.

[20] Faizi N, Kazmi S (2017). Universal health coverage-There is more to it than meets the eye. J Family Med Prim Care.

[21] Kamau MW, Mirie W, Kimani S (2018). Compliance with Iron and folic acid supplementation (IFAS) and associated factors among pregnant women: results from a cross-sectional study in Kiambu County, Kenya. BMC Public Health.

[22] Mulugeta A, Tessema M, H/Sellasie K, Seid O, Kidane G, Kebede A (2015). Examining Means of Reaching Adolescent Girls for Iron Supplementation in Tigray, Northern Ethiopia. Nutrients.

[23] Wiradnyani LA, Khusun H, Achadi EL, Ocviyanti D, Shankar AH (2016). Role of family support and women's knowledge on pregnancy-related risks in adherence to maternal iron-folic acid supplementation in Indonesia. Public Health Nutr.

